# Theory-Based, Participatory Development of a Cross-Company Network Promoting Physical Activity in Germany: A Mixed-Methods Approach

**DOI:** 10.3390/ijerph17238952

**Published:** 2020-12-01

**Authors:** Carina Hoffmann, Gerrit Stassen, Andrea Schaller

**Affiliations:** 1 Working Group Physical Activity-Related Prevention Research, Institute of Movement Therapy and Movement-Oriented Prevention and Rehabilitation, German Sport University Cologne, Am Sportpark Muengersdorf 6, 50933 Cologne, Germany; g.stassen@dshs-koeln.de (G.S.); a.schaller@dshs-koeln.de (A.S.); 2 Institute for Occupational Health Promotion, Neumarkt 35-37, 50667 Cologne, Germany

**Keywords:** workplace health promotion, physical activity, multicomponent intervention, micro- and small sized companies, cross-company networks, mixed-methods

## Abstract

The untapped potential of workplace health promotion (WHP) in smaller companies and the promising approach to promote physical activity in the workplace requires application-oriented approaches. This study describes the participatory, theory-based development of a cross-company network with a multicomponent intervention for promoting physical activity in smaller companies. The BIG-Manual (from the “Movement as an Investment for Health” project, German—BIG) was the theoretical framework for developing the cross-company network. Qualitative and quantitative data sources were used to identify the requests and requirements of stakeholders (employees on site, local exercise providers, company representatives and network partners) regarding measures promoting physical activity and the cross-company network. The methods applied included two workshops (n = 13; n = 15), individual semi-structured interviews (n = 8) and a survey (n = 285). The analysis revealed that a large number of stakeholders must be taken into consideration for physical activity promotion in cross-company networks. Many similarities between the requests of employees and further stakeholders concerning a multicomponent intervention for promoting physical activity could be identified. Present gender-specific and physical activity-related differences show the importance of target group-specific intervention planning in the context of WHP. This study makes an important contribution for the development of future cross-company networks promoting physical activity and yields valuable information for the design of a multicomponent intervention promoting physical activity.

## 1. Introduction

The health benefits of physical activity are well known and physical activity plays a significant role in the prevention of diseases [1,2,3,4,5]. Among others, physical activity is a significant protective factor for chronic diseases [1,6]. Considering the individual and socio-economic consequences associated with chronic diseases [7], the importance of physical activity promotion is beyond discussion. For adults, the World Health Organization (WHO) recommends at least 150 min of moderate-intensity aerobic physical activity or 75 min of vigorous-intensity per week [3]. Furthermore, major muscle groups should be strengthened at least two days a week [3]. Nevertheless, around 75% of adult men and 80% of adult woman in Germany do not meet the given recommendations of aerobic physical activity in combination with muscle strengthening made by the WHO [8]. Therefore, several strategies and measures promoting physical activity have been developed and implemented in Germany [9].

To promote a physically active lifestyle, the workplace seems to be a promising setting [2,10,11,12]. Previous research has indicated that measures promoting physical activity in the workplace can generate significant improvements in health, reduce absenteeism, psychological well-being and sick leave [13,14,15,16,17]. Furthermore, there are some indications of an increase in the level of physical activity due to workers’ participating in such measures [12]. Multicomponent interventions comprising different approaches and strategies to promote physical activity seem to be especially effective [2,13,18]. Within the context of behavioural-related interventions, the utilization of physical activity-related measures in the German population is clearly higher than the utilization of measures for nutrition and relaxation, whereby participation is less for men than for women [19]. Therefore, the literature refers to the importance of gender-sensitive physical activity promotion [20] and also a target group-specific orientation in workplace health promotion (WHP) [21].

Nevertheless, WHP still seems to be a challenging topic for companies. Thereby, less WHP is found especially in smaller companies [22,23,24,25,26,27,28]. Despite the fact that in Germany, about 96% of companies have a maximum of 49 employees [29], only 24% of health promotion programmes are realized in companies with this size [30]. The reasons for this can be different: for example lack of time and human resources or additional costs of these programmes [31,32]. To address this problem, the National Association of Statutory Health Insurance Funds in Germany decided to promote WHP in micro- and small-sized companies by founding cross-company networks [33]. This approach was introduced in 2014 in the German guideline on prevention [33]. In these cross-company networks, several companies are jointly advised on WHP. In this way, those networks can help to support companies that do not have enough resources for WHP [33], for example due to offering health promotion measures across the companies. Through regular cross-company exchange, the networks enable a low-threshold access to knowledge about WHP [33]. Local stakeholders such as representatives of social insurance can be involved as network partners in order to raise awareness for health issues [33]. The approach has led to the fact, that micro- and small-sized companies making up a share of around 39% of cross-company networks in 2018—about 15% more than by individual support by health insurance funds [30]. However, this approach has so far pursued the implementation of a holistic WHP in the companies in which physical activity is not the main focus. Furthermore, smaller companies have been a neglected setting to announce the potential of physical activity and physical activity promotion by now [24].

Due to the untapped potential of WHP in smaller companies and the promising approach of promoting physical activity in the workplace, the aim of this study is the participatory, theory-based development of a cross-company network for promoting physical activity. The research questions assessed include the following: (1) What are stakeholders’ requests with regard to physical activity-related measures and cross-company networks? (2) What are employees’ requests and requirements regarding physical activity-related measures? (3) What does the final multicomponent intervention for promoting physical activity look like?

## 2. Materials and Methods

From 2019 to 2022, the Federal Ministry of Health (BMG) is funding 10 projects under the “Exercise and the Promotion of Physical Activity” priority funding programme. Based on the background described, the present project was funded (the KomRueBer Project). The objective of KomRueBer is the development of a guideline for the conception and implementation of cross-company networks promoting physical activity. This paper describes the first step (conception phase), from July 2019 to March 2020 (9 months), with the development of the cross-company network and the multicomponent intervention for promoting physical activity.

### 2.1. Theoretical Framework

The BIG-Manual [34] was the theoretical framework for developing the cross-company network. It resulted from the “Movement as an Investment for Health” project (German—BIG) and it describes a participatory approach for promoting physical activity, for socially disadvantaged women [35]. Included phases of the BIG-Manual [34] are: (A) finding, (B) preparation, (C) cooperative planning process, (D) intervention process, and (E) ensuring sustainability.

In the present study, the participatory development of the cross-company network comprised phases (A), (B) and (C) and thus represented the conception phase, which lasted 9 months. Phase (D) and (E) form the implementation phase, which will immediately follow the conception phase and take 2 years to complete.

**Figure 1:**
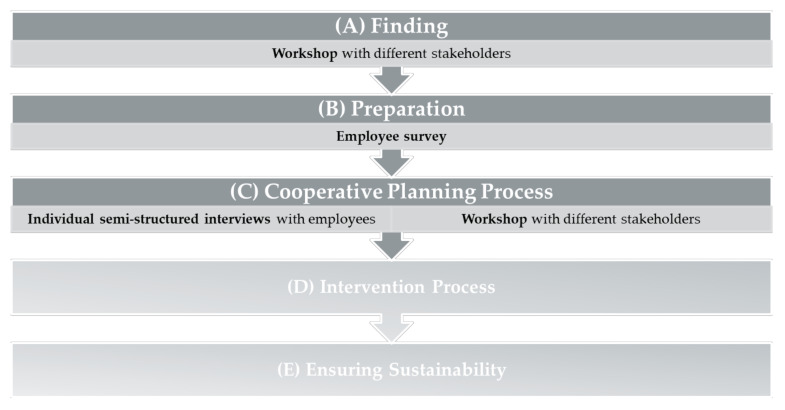
Methodical approach of the study within the framework of the BIG-Manual [34].

The research questions were answered by a mixed-methods design combining quantitative and qualitative data sources (see Figure 1). During (A) finding and (C) cooperative planning process, a qualitative research design was applied. The methods included workshops and individual semi-structured interviews with different stakeholders. A quantitative research design was applied during (B) preparation, and data were collected through an employee survey.

### 2.2. Setting and Study Population

The study was conducted in a technology park in Germany with around 90 different-sized companies. In this paper the definition of the size of companies—provided by the European Commission—is used [36]. By medium-sized companies, the European Commission [36] means companies with fewer than 250 employees. A small-sized company employs fewer than 50 persons, whereas a micro-sized company employs fewer than 10 [36].

The study population comprises different stakeholders: employees on site, local exercise providers, company representatives and network partners from public, economy, society/politics. Data were gathered in the period from July 2019 to February 2020.

Ethical clearance for the study was given by the German Sport University Cologne Ethics Committee (reference number: 120/2019).

### 2.3. Study Design

#### 2.3.1. Qualitative Study Design (A) Finding

• Stakeholder Workshop July 2019

In July 2019 a first workshop with company representatives, network partners from public, economy, society/politics, and exercise providers was realized (see Figure 1). The participating stakeholders were invited by e-mail, sent by the responsible manager of the cross-company network. The workshops lasted 2 h and took place in the technology park. The group discussions were conducted in German.

The aim of the first workshop was to understand the attitude of the stakeholders to physical activity-related measures and the cross-company network. Three key questions provided the basis for discussion: (a) “What could we offer in the technology park to promote physical activity?”; (b) “From your point of view, which other partners should we consider in the project?”; (c) “What has to happen so that the project is a success for you?”.

One moderator, two co-moderators for group discussions and one note-taker lead the first workshop. Each question was written down on a presentation board and presented by one of the moderators. The stakeholders were divided into three groups of equal size and discussed the questions station by station, whereby the moderators documented the feedback on the board. Feedback to question (a) was subdivided into the categories “exercise programmes”, “redesigning work processes” and “creating a physical activity-friendly infrastructure at work”, based on the tripartite in the National Recommendations for Physical Activity and Physical Activity Promotion (German—NEBB) [2]. During the workshop, handwritten field notes were also taken.

#### 2.3.2. Quantitative Study Design (B) Preparation

• Employee Survey

The employee survey was based on the results of the first workshop. Quantitative data were collected anonymously using the online survey tool EFS Survey (Questback GmbH) between October 16 and November 6 2019. The survey was addressed to all 2000 employees in the technology park. Participation was preceded by consent to the conditions described (via check box). The sample was obtained by sending an information sheet with a link and a QR-Code to the questionnaire to local company representatives. They were asked to forward the link and the information sheet to their employees. The sheet included short information about the purpose of the study and information about the content, the duration of the survey and the anonymity. In addition, the information sheets were hung up on site.

The survey comprised 25 target-group-specific questions. Table 1 shows the questions and answer categories for assessing the requests of the employees in relation to physical activity-related measures.

Furthermore, two questions inquiring for the motives and barriers of the employees for participating in physical activity measures in the workplace, one question inquiring about the willingness to participate in measures outside of working hours, and one question regarding the preferred intervention times were posed.

The following socio-demographic characteristics were assessed: gender (male; female; other), age (years), height (cm), weight (kg), occupational position (with or without management responsibility), education level (without graduation; secondary school qualification, secondary school certificate, 10-class general educational polytechnic secondary school; higher education entrance qualification, advanced technical certificate; another school leaving certificate, namely…), employment (full-time with a weekly working week of 35 h or more; part-time with a weekly working week of 15 to 34 h; part-time or by the hour with a weekly working time of less than 15 h; trainee, apprentice, “re-trainee”), family status (single; partnership/married with no child; partnership/married with child; single parent with child). For educational level and employment, standardised questions were used [37]. Furthermore, personal-related variables were collected. These included: type of shift work (no shift work; two shifts (early and late shift); three shifts (early, late and night shift), size of the company (≤9 employees; 10 to 49 employees; 50 to 249 employees; ≥250 employees) and distance travelled to workplace (km) (questions 9 to 19).

In addition, the physical activity of the participants was gathered (questions 20 to 24). For this purpose, the physical activity-related questions of the German Health Interview and Examination Survey for Adults (DEGS) [38] and two questions (muscle strengthening and work activity) of the German validated version of the European Health Interview Survey—Physical Activity Questionnaires (EHIS-PAQ) [39] were used. The questions of the EHIS-PAQ were taken from the questionnaire of the study “German Health Update” (GEDA 2014/2015-EHIS) of the Robert Koch Institute, which may be reused for scientific purposes [40]. The questionnaire closed with a question about employees’ desires and suggestions concerning the project (question 25).

• Statistical Analysis

Descriptive analysis (means (mean), standard deviations (±SD), frequencies (n) and percentages (%)) were conducted on all dimensions pertaining the requests of employees in relation to physical activity-related measures. For the description of the results, the three most-quoted answers referred to each dimension were considered. Descriptive analyses were also carried out for the socio-demographic parameters, the personal-related variables and the attainment of the physical activity recommendations. Missing data were not included. For the sample description, means and standard deviations (mean ± SD) were calculated for continuous data and frequency tables (*n*; %) for categorical data. In order to obtain more detailed information concerning the design of the multicomponent intervention, additionally subgroup-specific differences were examined in more detail. For this purpose, gender- and physical activity-related differences (reaching or not reaching the WHO recommendations for physical aerobic activity and reaching or not reaching the WHO recommendations for muscle strengthening) were considered. Group differences were tested by using the Pearson Chi-squared test. Statistical significance was set at *p* < 0. 5 and all statistical analyses were run with IBM SPSS 26 (IBM Corp., Armonk, NY, USA).

#### 2.3.3. Qualitative Study Design (C) Cooperative Planning Process

• Interviews

Data were gathered through individual semi-structured interviews in the period from November 2019 to February 2020 by the first author (C.H.). Participants were employees of micro-, small- and medium-sized companies, located in the technology park. All interviews were conducted in German, carried out on site, anonymized by using a code and were audio-recorded after written consent. The interview guidelines were developed on the results of the survey. The aim of the interviews was to gain a deeper understanding of the design of the multicomponent intervention from the employees’ point of view. The leading question was: “From the employees’ point of view, how should the multicomponent intervention for promoting physical activity should be designed?”. Furthermore, the participants were asked to complete three short questions (gender, year of birth, size of company in which the participants are employed). Table 2 shows the main topics and key questions of the discussion guide.

Additional questions were applied to maintain the interview and to obtain more detailed information (for example “From your point of view, when should the activity take place?”; “How can we inform employees about our activities?”; “Can you give an example?”).

Transcriptions were taken according to the rules of Dresing and Pehl [41]. The transcriptions were double-checked and the transcripts were analysed according to structuring content analysis [42,43], which is comparable to the framework method [44]. Main categories based on the semi-structured interview guideline were generated and applied to the transcripts. In addition, the researchers examined the data for new themes and issues, so that new categories were formed if necessary. The categories and assigned text fragments were compared until a consensus was reached by the two researchers. For the analysis, the researchers used the MAXQDA 11 software. The quotations are marked with the respective participant code and the corresponding text passage.

• Stakeholder Workshop January 2020

The second stakeholder workshop lasted 4 h, also took part in the technology park and the invitation process was analogous to that of the first workshop. As in July 2019, company representatives, network partners from public, economy, society/politics, and exercise providers participated.

The aim of the second workshop was to pre-finalize the multicomponent intervention for promoting physical activity. Based on the results of the employee survey and first impressions of the semi-structured interviews (see Figure 1) the stakeholders discussed the following questions: (a) “How can the environmental measures be implemented?”; (b) “Which specific physical activity measures can you provide?”; (c) What types of physical activity do you already offer in your company? Which of these could be opened for the other companies?”. The network partners from economy discussed question (a), the networks partners from the public, society/politics as well as the exercise provider question (b) and the company representatives question (c). The discussions were documented on the presentation boards and transferred to an action plan. Handwritten field notes—taken during the workshop—completed the documentation.

## 3. Results

### 3.1. Stakeholder Requests Regarding Physical Activity-Related Measures and the Cross-Company Network

#### Stakeholder Workshop/(A) Finding

Thirteen people (10 female, 3 male) participated in the first stakeholder workshop: two exercise providers, seven company representatives and four network partners (two “economy” and two “society/politics”). Within the scope of the project, three participants had a double role (once: network partner and exercise provider; twice: company representatives and network partner). Ideas for physical activity showed the subcategories: “exercise programmes”, “redesigning work processes” and “creating physical activity-friendly infrastructure at work” (see Table 3).

The requests, focused on “exercise programmes”, were multifaceted. They included inter alia individual consulting and courses, events, team sports or gamification approaches. Regarding the requests concerning “redesigning work processes”, the stakeholders noted opportunities to make physical activity a routine in everyday work: for example, through physical activity breaks in the morning, ergonomic adjustment or flexible working hours. In order to “create physical activity-friendly infrastructure”, stakeholders mentioned possibilities for training (indoor/outdoor) and further facilities like showers or bicycle stands.

With regard to the integration of *further partners* in the network, the participants suggested two local companies and various regional exercise providers (e.g., fitness studios, bike-leasing providers, local sports clubs) as well as the city administration, local media and company doctors.

From the stakeholders’ point of view, transparent communication about the activities was seen as a key *success factor* for the project. It also became clear that someone is needed to continuously take care of the topic. From the company’s perspective, increasing attractiveness as an employer, due to participating in the cross-company network, was seen as a further success factor, whereas network partner and exercise providers hoped for major local awareness from participating in the project.

### 3.2. Employee Requests and Requirements Regarding Physical Activity-Related Measures

#### Survey/(B) Preparation

Two hundred and eighty-five employees (female: 164 (58.2%); mean age: 36.6 (±10.3)) participated in the survey. Baseline characteristics of the sample are shown in Table 4. 57 (20.0%) of the participants met the WHO recommendations for physical aerobic activity, and 77 (27.0%) met the WHO recommendations for muscle strengthening.

Table 5 shows the main requests of the employees with regard to physical activity-related measures.

With regard to the content of the interventions, the employees particularly wanted information about exercises for certain complaints (e.g., back pain) (53.0%), information about muscle-strengthening (52.6%), and compensatory exercises for the working day (51.2%). To become more physically active at work, about 50% of the participants described instructed physical activity breaks and an office organization, that supports movement, as beneficial. Concerning to the infrastructure, more than the half of the attendees asked for a fitness room (68.4%), showers (58.6%) and a locker room (57.2%). With regard to the formats gym courses (61.1%), outdoor activities (55.8%) and job-related programs (40.0%) were requested in particular.

Gender-specific statistical differences—concerning the most selected answer options—could be shown four times (“environmental conditions”: instructed physical activity breaks (female: 94 (57.3%), male: 43 (36.8%); *p* value = 0.001); “infrastructure”: showers (female: 87 (53.0%), male: 79 (67.5%); *p* value = 0.015); “format”: gym courses (female: 123 (75.0%), male: 51 (43.6%); *p* value < 0.001); job-related programmes (female: 78 (47.6%), male: 36 (30.8%); *p* value = 0.005).

Participants who reached the WHO recommendations for physical aerobic activity and participants who did not reach them differentiate concerning the requests two times (“environmental conditions: people motivating me to exercise” (reaching WHO recommendations for physical aerobic activity: 11 (19.3%), not reaching WHO recommendations for physical aerobic activity: 99 (43.8%); *p* value = 0.001)); “infrastructure: showers” (reaching WHO recommendations for physical aerobic activity: 42 (73.7%), not reaching WHO recommendations for physical aerobic activity: 125 (55.3%); *p* value = 0.012).

With regard to the subgroups “reach the WHO recommendations for muscle strengthening” and “do not reach the WHO recommendations for muscle strengthening”, statistically significant differences were found once (“environmental conditions: people motivating me to exercise” (reaching WHO recommendations for muscle strengthening: 19 (24.7%), not reaching WHO recommendations for muscle strengthening: 91 (44.2%); *p* value = 0.003)).

### 3.3. Multicomponent Intervention for Promoting Physical Activity

#### 3.3.1. Qualitative Interviews/(C) Cooperative Planning

**Figure 2:**
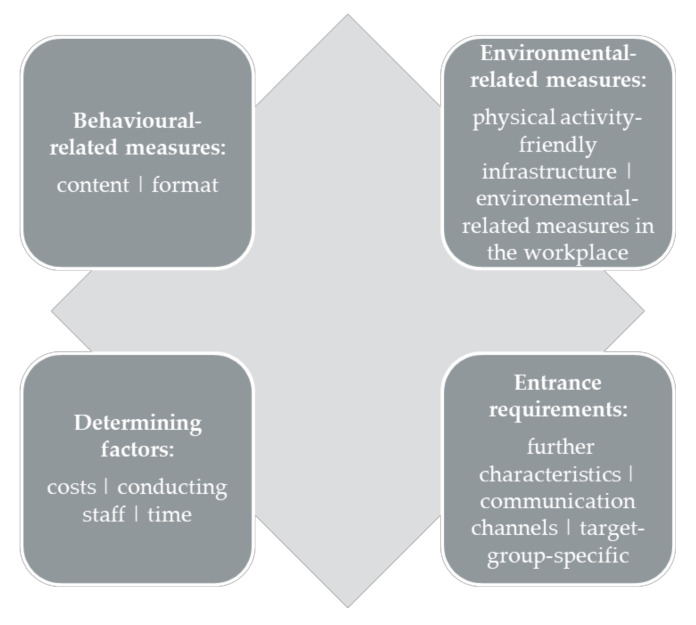
Main topics.

The sample consisted of 3 female and 5 male participants and the age ranged between 20 and 63 (mean 33 ± 14). The interviews lasted from 30 to 60 min and the average duration of the interviews was 43 (±14) min. The interview-data revealed requests regarding four main topics (behavioural-related measures, environmental-related measures, determining factors and entrance requirements), divided into further subcategories (see Figure 2).

• Behavioural-Related Measures

Employees particularly expressed their interest in the **content** of muscle strengthening. The data indicated that there was also a desire to receive information about exercises for certain complaints and targeted compensation. Furthermore, the participants were interested in stress regulation through physical activity. Regarding the desired **formats**, outdoor activities and courses were preferred.

*“So exercise to reduce stress would also interest me. And of course, it is clear, how you might do any exercise to create prevention (…). Sure, I have a sedentary job. There are certainly exercises that are useful to improve certain postures or something, because you keep catching yourself sitting in the chair and lounging in the chair. I think there are certain posture-related exercises that you can do that could prevent the whole thing”*

*(male employee, YK23C, 61).*

• Environmental-Related Measures

Statements referring to indoor **physical activity-friendly infrastructure** focused on “changing rooms”, “gyms” and “showers”. The general statements regarding outdoor infrastructural conditions were manifold. Secure bicycle stands, outdoor fitness trails and open spaces for team sports were mentioned. **Environmental-related measures** in the workplace focused on both measures that can incorporated into work processes and on ergonomic aspects in the workplace. Participants felt positive about instructed exercise breaks during the workday. However, it was assumed that it can be integrated into the workday and planned in advance.

*“So I think if I knew there is a large room somewhere, I would know there I can take a shower, I can take my bag with me, I can stow it safely there, I can change there, can take a shower and so on, then it would be that for me. So then I know that I would 100%, definitely use that”*

*(male employee, YK23C, 81).*

• Determining Factors

For the participants it was important that the measures to be **free of charge**, whereby to a certain extent there was the willingness to pay a small contribution. In addition, qualified **conducting staff** were considered as highly relevant. In particular, with regard to the use of equipment made available, reference was made to the need for professional guidance and instruction. Concerning the intervention **times,** most of the participants would like to participate in a physical activity measure after working hours. The specific desired start time varied, depending on the individual workday.

*“I think it is very important that it is a free offer. Or maybe, if it is really a very regular one and you use the infrastructure, a low contribution. But keep the inhibition level as low as possible so that you accept it at all”*

*(female employee, KN46C, 77).*

• Entrance Requirements

In consideration of **further characteristics**, statements made clear that a low-threshold integration into the workday should be ensured and is a benefit of physical activity measures in the workplace. Age was mentioned in the context of **target-group specificity**. The participants requested measures that take their individual age into account. With regard to the **communication channels**, the interviewees preferred a website that informs about the measures and allows registration. The statements regarding the utility of e-mail and newsletters were manifold. Referring to analogous media, the participants frequently mentioned posters and notices as a good opportunity.

*“So, from my personal point of view, you would have to make sure that the measures are presented in such a way that they can be integrated into everyday work, maybe a lunch break”*

*(male employee, YK23C, 73).*

#### 3.3.2. Stakeholder Workshop/(C) Cooperative Planning

As part of the cooperative planning (C) (see Figure 1) 15 persons (11 female and 4 male) participated in the second stakeholder workshop. Again exercise providers (n = 2), company representatives (n = 6) and network partners (n = 7: three “economy” and four “society and politics”) took part in the workshop and the three participants with double roles were present.

• Multicomponent Intervention

**Figure 3:**
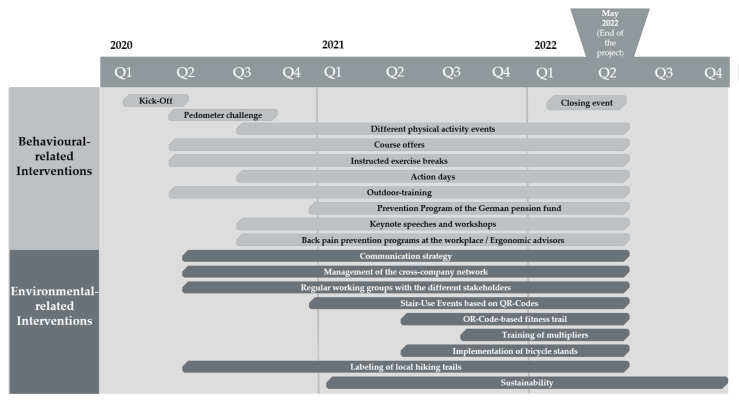
Overview and timeline of the multicomponent intervention. Q: quarter.

Figure 3 illustrates the final multicomponent intervention for the cross-company network, shown as a timeline for implementation.

The framework for the multicomponent intervention is formed by a kick-off at the beginning of the implementation phase (March 2020) and a closing event at the end of the project (May 2022). A pedometer challenge will follow immediately after the kick-off. Several courses and outdoor measures, the content of which is based on the requests, as well as instructed exercise breaks during the lunchtime, will take place continuously over the project period. To promote the collaboration of different German social insurance companies, a prevention programme of the German pension fund will be integrated. Back pain prevention programmes will be offered and ergonomic advisors will tutor the employees directly in the workplace. At the infrastructural level, the implementation of bicycle stands and the labelling of local hiking trails shall be realized. QR-Code-based measures to promote stair use and a QR-Code-based fitness trail are further activities to establish on site. By scanning the QR-Codes, the user will be lead to different videos, which screen physical activity exercises. These exercises can be performed in the stairwell or outdoors (e.g., on a park bench). For this purpose, the QR-Codes will be installed in the stairwells and in form of a fitness trail (outdoors). Near the end of the project, multipliers will be trained. Multipliers are employees who will be educated to offer short exercise breaks for their colleagues in the workplace. Overall, the implementation of a professional communication strategy for physical activity promotion (including a web-based platform) is planned. On the organisational level, working groups with different stakeholders will take place regularly to discuss the status of the cross-company network and the multicomponent intervention. A network manager will be responsible for overall coordination. The elements mentioned will be available to all employees in the technology park, regardless of the employment with a company. They will be offered across the companies so that every company has the opportunity to let its employees participate. The local exercise providers will realize the measures on site and the health insurance provider will provide financial support.

## 4. Discussion

The multicomponent intervention, developed in a participatory approach, includes behavioural and environmental measures to promote physical activity over 26 months.

The present results initially confirm the need for measures to promote physical activity among the target group. As only 20% of the employees participating in the survey met the WHO recommendations for aerobic physical activity [3] and 27% the recommendations for muscle strengthening [3], the need for the multicomponent intervention is beyond discussion. Regarding aerobic physical activity, the present results are, therefore, well below the results of population based-surveys in Germany (45.3% [8]). Female employees achieved the recommendations less often than the male employees, which corresponds to previous surveys [8,46]. The fact that the majority of those questioned are predominantly sedentary, confirms once again the need for physical activity measures on site.

Regarding the network development, the participants of the stakeholder workshop (A) suggested numerous other partners, e.g., clubs, fitness studios, commercial providers and the municipality. This mainly confirms the results of the SAMBA (German acronym—Systematic recording of relevant actors in the promotion of physical activity) study [47,48] and other projects and recommendations [34,47,48,49], showing that relevant actors in physical activity promotion can come from a variety of sectors of society. However, it was noticeable that in our results, compared to existing studies [47,48], actors from the social sector “health” were hardly mentioned as potential project partners. Only company doctors were mentioned in this context, which could be related to the fact that a health insurance company and a therapy centre were already involved and participated in the stakeholder workshop.

The requests of the participants towards physical activity measures in the workplace showed many parallels with previous research. Because of its effectiveness [2,13,18], in this study value was placed on multicomponent intervention design right from the beginning. Therefore the tripartite, incorporating measures of “exercise programmes”, “redesigning work processes” and “creating physical activity-friendly infrastructure at work”, analogous to the National Recommendations for Physical Activity and Physical Activity Promotion [2], was assumed. Concerning “exercise programmes”, stakeholders and employees mentioned classic course measures as a favourable opportunity to promote physical activity, which goes in line with other studies [50,51]. With reference to a “physical activity-friendly infrastructure”, employees and at the stakeholder level especially company representatives stated that fitness rooms, showers and locker rooms are a good measure to promote physical activity behaviour on site. This correspondents to previous findings [50,51], where fitness centres were seen as a preferred measure for health promotion and showers with changing rooms an enabler for physical activity. Similarities with previous research could be also reported pertaining to the factor “redesigning work processes”. Our results show that a flexible timing of the measures, in work planning and easy access, were seen as important factors for participation at the organisational level, which goes in line with a number of several studies [52,53,54]. However, it was noticeable that over three-quarters of the employees favoured the measures after working hours, while far less than half could imagine during working hours. Parallels could be drawn here at the stakeholder level. While exercise providers and network partners mentioned physical activity during working hours, reference was only made to flexible working hours at the company level. This could be due to company-specific circumstances, which Zok [55] already assumed. Existing working hour models, the work activity as such or missing infrastructure could be issues. If employees considered the option for physical activity measures during working hours to be possible at all, they might also state and favour the option. Further, transparent communication about the measures was regarded as a key success factor for both stakeholders and employees. Person et al. [56] as well as Stummer et al. [57] emphasize the importance of marketing and communication within health promotion programmes. Finally, the employees mentioned the importance of free measures, to which Persson and colleagues [58] also refer.

Taken as a whole, our analysis revealed many similarities between the requests of local stakeholders and employees concerning a multicomponent intervention for promoting physical activity. On the employee level, overall, the qualitative findings underline the results of the survey. Present gender-specific and physical activity-related differences in relation to the requests of employees show the importance of targeting group-specific intervention planning in the context of WHP, which previous research has already indicated [21,59].

To sum up, our components of the final multicomponent intervention are regularly implemented in the context of WHP. Several of the intervention types have already been examined for their effectiveness, e.g., pedometer measures [60,61], physical activity breaks [14] or stair use [62]. However, it must be taken into account that the results on the effectiveness of individual measures are still inconclusive [12,63,64]. Rather, the strength must be seen in the multidimensional nature of our intervention approach, which is referred to in the literature [2,18,65]. In addition, care is taken to deploy appropriately qualified personnel in accordance with the prevention guidelines of the statutory health insurance [33]. By means of the communication strategy a strength-based approach to physical activity promotion, based on the research of Warburton and colleagues [66,67], is intended. As the literature also recommends [21], target group and gender-specific differences were considered in the intervention planning on the one hand, in relation to the individual parts of the multicomponent intervention, on the other hand in relation to different approaches and channels of communication. When developing the communication strategy, attention will be paid to use gender-neutral language or to address both genders. Both genders will be represented in an equal relation, for example when it comes to the personnel of trainers or the performer within the QR-code based videos. In order to satisfy the various needs related to the accessibility of the target groups, the physical activity measures will be offered at different times. This is to ensure that part-time employees, full-time employees and employees with less flexible working hours can participate in the measures. Some of the physical activity measures (e.g., back pain prevention programmes; ergonomic advises; instructed exercise breaks) will be realized directly at the workplace and during working hours. In this way, employees who otherwise would not take part in behavioural-related measures should be reached. Besides, the combination of behavioural- and environmental-related measures intends to reach target groups, which do not feel addressed by one of the approaches. A wide range of measures within the multicomponent intervention should meet the various needs and preferences of the target group. For requests that cannot be implemented for various reasons (e.g., costs, insurance aspects, maintenance requirements), alternatives were developed as part of the second stakeholder workshop and afterwards (e.g., QR-Code-based fitness trail instead of a fitness room).

### Strengths and Limitations

A major strength of the present study is the participatory involvement of the employees and stakeholders in the intervention planning. Based on the participation levels according to Wright [68], participation of those involved in the present study is extended to “partly discretionary competence”. Previous research valued the involvement of various stakeholders as a criterion of quality [65] and stated the importance of a participatory development of WHP programmes [69,70,71,72]. In the sense of participatory research, the individual success factors of the stakeholders concerning the project, and gathered within the first stakeholder workshop were considered as relevant outcomes for the evaluation approach. Another strength can be seen in the mixed-methods approach of this study. The applied combination of qualitative and quantitative research methods enabled a comprehensive insight into desired physical activity-related measures. The stakeholder workshop (A) facilitated an initial exploration of the issue and built the foundation for the development of the subsequent survey (B). This later allowed obtaining a comprehensive picture of the employees’ point of view, and due to the interviews (C) the results could be validated and more detailed information be obtained.

However, some limitations have to be stated. Only 285 of the total of around 2000 employees on site took part in the survey. Although a participation rate of about 14% is a common value in surveys, one cannot assume it to be a representative sample. Second, the study includes a small number of interviews with employees from only four companies. This can also limit the generalizability of the results. The group of interviewees was rather homogeneous and generally had a positive attitude towards the subject of exercise. Time-restricted resources did not allow for the implementation of further interviews. Overall, the entire sample was quite young and more women than men participated in the survey. It must also be taken into account that the study was carried out in an urban setting. Both points limit the generalization of the results. Furthermore, no chronic disease rates were covered in the target population.

## 5. Conclusions

In order to increase physical activity behaviour in the population and to give smaller companies the opportunity to implement appropriate measures, practitioners require application-oriented approaches. Our results show that a large number of stakeholders must be taken into consideration for physical activity promotion in cross-company networks, also outside the physical activity specialized field. At the same time this requires comprehensive cooperation with individual participants. We recommend broad involvement of stakeholders in order to achieve wide acceptance of the measures. Since physical activity promotion in the workplace has many facets and the settings include a heterogeneous target group, a wide range of measures is required in order to meet various needs. Considering the increasing amount of older workers and the fact that many industries are still characterized by more male employees, further research is recommended to gain more insights from all demographic groups. Likewise, the transfer to other regions requires a critical examination of the local conditions and possibilities of promoting physical activity within the framework of cross-company networks.

## Figures and Tables

**Table 1:** Non-standardised questions for assessing the requests in relation to physical activity-related measures (translation).

Dimension	Question	Answer Categories *
**Content**	What would you like to know about physical activity?	“How I can increase my endurance”, “How I can strengthen my muscles”, “How I can improve my flexibility”, “How I can reduce stress through exercise”, “Which movements or forms of exercise are suitable for certain complaints (e.g., back pain)”, “Which exercises I can incorporate to compensate for everyday work”, “How I can make my day-to-day work more physical activity-friendly”, “How I deal with one-sided burdens in the workplace”, “How I can overcome my weaker self”, “How can I lose weight”, “How I can improve my figure”, “Where I can find suitable physical activity options nearby”, “Others…”
**Environmental conditions**	What would help you become more physically active at work?	“Instructed physical activity breaks”, “Flexible workplaces (e.g., the possibility to work outside)”, “People motivating me to exercise”, “Office organization that supports movement (e.g., standing space)”, “Physical activity-friendly redesign of operational processes (e.g., holding meetings while standing instead of sitting)”,”That my supervisor knows more about physical activity”, “Others…”
**Infrastructure**	What other factors would help you to integrate more physical activity into your everyday work?	“Pedestrian-friendly design of the technology park (e.g., paths)”, “Bike-friendly design of the technology park”, “Establishment of a fitness room in the technology park”, “Fitness/exercise areas in the open air (e.g., “fitness trail”)”, “Motivating design of the stairwells in the technology park (e.g., stairs with slogan stickers)”, “Information on hiking trails in the area”, “Sufficient bicycle parking spaces”, “Sufficient e-bike charging stations”,” Establishment of an e-bike/bicycle pool for the technology park”, “Movement-friendly design of communal areas (e.g., with table tennis tables, table football)”, “Showers”, “Locker room”, “Others…”
**Format**	In which form should an intervention take place so that you are willing to participate?	“Individual consulting service”, “Lectures”, “Half-day workshop”, “Full day workshop”, “Half-day seminar”, “Full day seminar”, “Health days/action days”, “Movement challenges (e.g., pedometer competition)”, “Events (e.g., company runs)”, “Gym courses”, “Job-related programmes (e.g., back pain prevention)”, “Outdoor activities”, “Digital measures (e.g., via apps)”, “Team sport/company sport, if so which:…”, “Measures in other languages, namely…”, “Others…”

* Multiple answers were possible.

**Table 2:** Topics and key questions of the discussion guide (translation).

Topic	Key Question
**Behaviour-and environmental-related measures**	“What type of physical activity intervention would you be interested in?”
**Infrastructure**	“What would have to change about the infrastructure in the technology park in order to have a more physically active workday?”
**Determining factors and entrance requirements**	“How can our future measures be integrated well into your workday here in the technology park?”; “What would be important when designing the physical activity measures so that you would be motivated to do it?”

**Table 3:** Ideas of participating stakeholders for physical activity in the cross-company network (translation).

“What Could We Offer in The Technology Park to Promote Physical Activity?”		
Exercise Programmes	Redesigning Work Processes	Creating Physical Activity-Friendly Infrastructure at Work
**Exercise Providers**		
“Walk and Talk” (coaching combined with physical activity) Individual consulting Prevention courses Teambuilding and communication measures Brain jogging Gamification measures Balance training (balance board/trampoline) Cross-linking to local sport programmes	Physical activity during working hours Physical activity measures in the morning	Barefoot path Fitness parkour Climbing wall/sports park (Electric) Bicycle rental/workshop/bicycle stand
**Company representatives**		
Team sports Bicycle-events Corporate-events Charity-events Pedometer challenge Course measures (climbing/swimming)	Stairs instead of elevator Physical activity-promoting tools (e.g., pedal-trainer for the office) Flexible working hours Physical activity in the morning	Showers/locker rooms Equipment room Digital information platform (overview, prices, plans) Trampoline Swimming bath (Electric) Bicycle rental/workshop/bicycle stand
**Network partners**		
• No statements in this topic given	Flexible workstations Seek talks with employers: “How can we support you?” Regularly inform employers about the topic Consulting: “Physical activity in the working day” Physical activity during working hours	Pedestrian-friendly walkways (Electric) Bicycle rental/workshop/bicycle stand

**Table 4:** Sample characteristics.

Sample Characteristics	
Age (years) (n = 281) [mean (SD)]	36.6 (±10.3)
Sex (n = 282) [n; %]	
female	164 (58.2%)
male	117 (41.5%)
other	1 (0.3%)
Body mass index (BMI) (n = 265) [mean (SD)]	24.7 (± 4.2)
Classification ^1^ (n = 265) [n; %]	
Underweight (<18.5)	11 (4.2%)
Normal weight (18.5–24.9)	151 (57.0%)
Pre-obesity (25.0–29.9)	71 (26.8%)
Obesity class I–II (30.0 – 39.9)	32 (12.1%)
Highest Level of education (n = 279) [n; %]	
“secondary school qualification”	23 (8.2%)
“higher education entrance qualification/advanced technical certificate”	231 (82.8%)
“another school leaving certificate”	25 (9.0%)
Employment (n = 280) [n; %]	
“full-time employed; ≥35 h/week”	243 (86.8%)
“part-time employed; 15-34 h/week”	30 (10.7%)
“part-time or by the hour; ≤15h/week”	1 (0.4%)
“trainee, apprentice, re-trainee”	6 (2.1%)
Size of the Company (n = 280) [n; %]	
“≤9 employees”	12 (4.3%)
“10–49 employees”	10 (3.6%)
“50–249 employees”	106 (37.9%)
“≥250 employees”	152 (54.3%)
Work activity (n = 283) [n; %]	
“mainly sedentary/standing”	256 (90.5%)
“mainly walking/moderate physical activity”	24 (8.5%)
“mainly heavy manual work”	2 (0.7%)
“no work-related activities”	1 (0.4%)
World Health Organization (WHO) recommendations for physical aerobic activity (n = 283) [n; %]	
“meeting the WHO recommendations for physical aerobic activity”	57 (20.1%)
“not meeting the WHO recommendations for physical aerobic activity”	226 (79.9%)
WHO recommendations for muscle strengthening (n = 283) [n; %]	
“meeting the WHO recommendations for muscle strengthening”	77 (27.2%)
“not meeting the WHO recommendations for muscle strengthening”	206 (72.8%)

SD, standard deviation; ^1^ [45].

**Table 5:** Requests with regard to physical activity-related measures (translation).

Dimension (Question)	Main Results (Top Three of the Selected Answer Options) *	Sample (n = 285) [n; %]	Female (n = 164) [n; %]	Male (n = 117) [n; %]
**Content** (“What would you like to know about physical activity?”)	• Exercises for certain complaints	151 (53.0%)	96 (58.5%)	55 (47.0%)
	• Muscle strengthening	150 (52.6%)	90 (54.9%)	59 (50.4%)
	• Compensatory exercises for the working day	146 (51.2%)	87 (53.0%)	58 (49.6%)
**Environmental conditions** (“What would help you to get more physical active at work?”)	• Instructed physical activity breaks	138 (48.4%)	94 (57.3%)	43 (36.8%)
	• Office organization that supports movement	137 (48.1%)	85 (51.8%)	51 (43.6%)
	• People motivating me to exercise	110 (38.6%)	66 (40.2%)	43 (36.8%)
**Infrastructure** (“What other factors would help you to integrate more physical activity into your everyday work?”)	• Fitness room	195 (68.4%)	108 (65.9%)	85 (72.6%)
	• Showers	167 (58.6%)	87 (53.0%)	79 (67.5%)
	• Locker room	163 (57.2%)	90 (54.9%)	73 (62.4%)
**Format** (“In which form should an intervention take place so that you are willing to participate?”)	• Gym courses	174 (61.1%)	123 (75.0%)	51 (43.6%)
	• Outdoor activities	159 (55.8%)	96 (58.5%)	62 (53.0%)
	• Job-related programmes (e.g., back pain prevention)	114 (40.0%)	78 (47.6%)	36 (30.8%)

* Multiple answers were possible (compare Table 1).
